# Exploring the Validity of GOLD 2023 Guidelines: Should GOLD C and D Be Combined?

**DOI:** 10.2147/COPD.S430344

**Published:** 2023-10-24

**Authors:** Christopher Duckworth, Michael J Boniface, Adam Kirk, Thomas M A Wilkinson

**Affiliations:** 1 IT Innovation Centre, Digital Health and Biomedical Engineering, University of Southampton, Southampton, UK; 2 My mHealth Limited, London, UK; 3 National Institute for Health Research Biomedical Research Centre, University of Southampton, Southampton, UK; 4 Faculty of Medicine, University of Southampton, Southampton, UK

**Keywords:** Global Initiative for Chronic Obstructive Lung Disease, differential disease progression, exacerbation rate

## Abstract

**Introduction:**

The GOLD (Global Initiative for Chronic Obstructive Lung Disease) 2023 guidelines proposed important changes to the stratification of disease severity using the “ABCD” assessment tool. The highest risk groups “C” and “D” were combined into a single category “E” based on exacerbation history, no longer considering symptomology.

**Purpose:**

We quantify the differential disease progression of individuals initially stratified by the GOLD 2022 “ABCD” scheme to evaluate these proposed changes.

**Patients and Methods:**

We utilise data collected from 1529 users of the myCOPD mobile app, a widely used and clinically validated app supporting people living with COPD in the UK. For patients in each GOLD group, we quantify symptoms using COPD Assessment Tests (CAT) and rate of exacerbation over a 12-month period post classification.

**Results:**

CAT scores for users initially classified into GOLD C and GOLD D remain significantly different after 12 months (Kolmogorov–Smirnov statistic = 0.59, P = 8.2 × 10^−23^). Users initially classified into GOLD C demonstrate a significantly lower exacerbation rate over the 12 months post classification than those initially in GOLD D (Kolmogorov–Smirnov statistic = 0.26; P = 3.1 × 10^−2^; all exacerbations). Further, those initially classified as GOLD B have higher CAT scores and exacerbation rates than GOLD C in the following 12 months.

**Conclusion:**

CAT scores remain important for stratifying disease progression both in-terms of symptomology and future exacerbation risk. Based on this evidence, the merger of GOLD C and GOLD D should be reconsidered.

## Introduction

Chronic obstructive pulmonary disease (COPD) is a common, and incurable respiratory disease affecting over 170 million people worldwide.^1^ The Global Initiative for Chronic Obstructive Lung Disease (GOLD) aims to guide therapy through a proactive approach to determining disease severity.^2^ The GOLD 2023 guidelines proposed important changes to the stratification of disease severity using the ABCD assessment tool.^3^ The highest risk groups “C” and “D” were combined into a single category “E” based purely on exacerbation history, no longer stratifying further on symptomology. This letter evaluates the proposed change to GOLD guidelines by considering the differential disease progression of individuals initially stratified by the GOLD 2022 (ABCD) scheme. For each group, we quantify symptoms using COPD Assessment Tests (CAT) and rate of exacerbation over a 12-month period post classification in a UK-based COPD cohort.

## Materials and Methods

**Figure 1:**
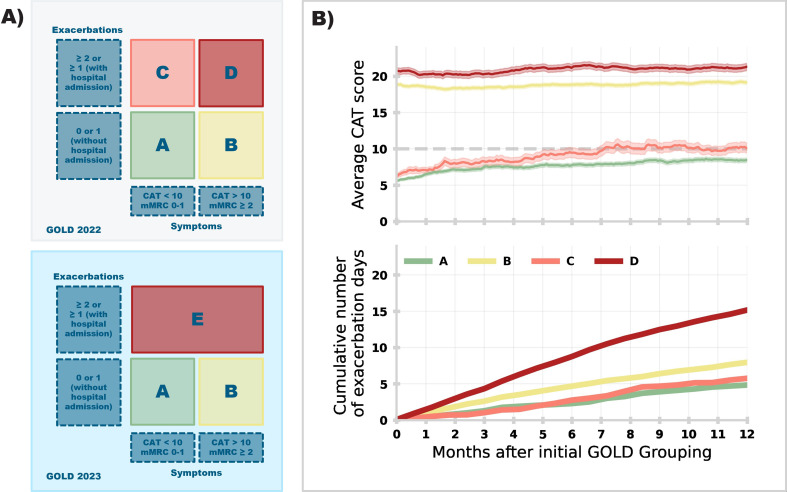
(**A**) Changes to GOLD group guidelines from 2022 (top) to 2023 (bottom). (**B**) Average CAT score (top) and average cumulative number of self-reported exacerbation days (bottom) registered in the 12-months post initial GOLD 2022 grouping. An exacerbation day is defined as a unique day where a given user has reported a moderate or severe exacerbation in their daily symptom scores registered in-app.

We utilise data collected from the myCOPD mobile app, a widely used and clinically validated app supporting people living with COPD in the UK.^4–6^ All users of the myCOPD app are clinically diagnosed with COPD, with app usage limited to patients “prescribed” the app by clinicians as part of agreed care plans. myCOPD facilitates self-management of COPD through educational content, localised weather and pollution levels, and digital diaries for users to keep track of medications and symptoms. As part of their onboarding, users submit an annual report of their exacerbation history and a CAT score which is used to stratify patients into GOLD groups (Figure 1A) to help clinicians make treatment decisions. Users also register a Modified Medical Research Council (mMRC) Dyspnea score; however, CAT is the primary base for stratification. We define exacerbation events as an acute sustained worsening of a patient’s condition that requires rescue medication (ie, moderate) or emergency care (ie, severe), including hospitalisation.^7^

Users self-report disease progression through symptom reporting (daily symptom reports on 4-point scale,^8^ CAT,^9^,^10^ mMRC) and exacerbations (self-report of emergency medicine or care requirement in symptom scores, annual retrospective report of exacerbations). We follow COPD disease progression as marked by symptomology and exacerbation rate for 1529 users who frequently register self-reported information over 12 months post onboarding and initial stratification into GOLD 2022 gold groups (A: 333, B: 830, C: 91, D: 275 users). Selection criteria included having a CAT score and an annual retrospective report of exacerbation history at baseline and having at least five further CAT scores registered over the following 12-month period.

We evaluate the likelihood of two cohorts of being drawn from the same continuous distribution through implementation of a two-sample Kolmogorov–Smirnov (KS) test. A high KS value combined with a low p-value (eg, KS ≥ 0.1 for p ≤ 0.01 confidence level) is consistent with the two samples being statistically significantly different. Averages are presented as a mean over the GOLD group.

The study data was originally collected by my mHealth Ltd using the myCOPD app. Consent for the original data collection is held by my mHealth Ltd, and the app users (the data subjects) would expect their data to be used for ethically approved research. The my mHealth Privacy Notice^11^ contains the wording: “Healthcare & research teams. This will always be anonymised unless you agree, at the time, to participate in trials using your identifiable information”. This study was conducted as secondary data analysis by the University of Southampton with the legal basis to process special category personal data in accordance with UK GDPR Article 6 (e) “Public Task” and UK GDPR Article (h) “Health or social care (with a basis in law)” and within scope of Data Protection Act 2018 Article 2 “Health or social care purposes”. The study received ethics approval from the University of Southampton’s Faculty of Engineering and Physical Science Research Ethics Committee (ERGO/FEPS/66535) and was reviewed by the University of Southampton Data Protection Impact Assessment panel, with the decision to support the research.

## Results

We calculate the average CAT score for all 1529 users (Figure 1B, top). We also compute the average cumulative number of exacerbation days for 690 users who registered regular symptom scores in the 12-month period post initial stratification (Figure 1B, bottom). An exacerbation day is when a user has reported a moderate or severe exacerbation (ie, emergency medication or care required respectively).

Users initially classified as GOLD C show the quickest progression of symptoms with the average CAT score rising to ~10 from 7 months post classification. About 25.2% of GOLD C users would be re-classified as GOLD D after 12 months (relative to 32.0% of initial GOLD B users). CAT scores for users initially classified into C and D remain significantly different after 12 months (Kolmogorov–Smirnov statistic = 0.59, P = 8.2 × 10^−23^). Users initially in GOLD D show a higher cumulative total of exacerbation days (over 12 months post classification) than all other groups, with initial GOLD B having a higher rate than C.

Table 1 provides the estimated rate of moderate (ie, emergency medication required) and severe (ie, emergency care required) exacerbations in the 12 months post initial GOLD classification. Estimation is based on an annual retrospective report submitted by 943 users (61.9% of cohort) collected at least 12 months after initial GOLD classification. Users initially classified into GOLD C demonstrate a significantly lower exacerbation rate over the 12 months post classification than those initially in GOLD D (Kolmogorov–Smirnov statistic = 0.26; P = 3.1 × 10^−2^; all exacerbations). Further, those initially classified as GOLD B have higher moderate and severe exacerbation rates over the 12 months post classification than those initially in GOLD C.

**Table 1:** Average Rate of Moderate and Severe Exacerbations Over 12 Months Post GOLD Group Classification. Errors are Given by Standard Error on the Mean

	Initial Gold Group			
	A	B	C	D
**Average number of moderate exacerbations over 12 months post classification**	0.757 [±0.119]	1.850 [±0.095]	1.237 [±0.273]	2.296 [±0.210]
**Average number of severe exacerbations over 12 months post classification**	0.068 [±0.025]	0.209 [±0.032]	0.053 [±0.037]	0.376 [±0.078]

## Discussion

The 2023 GOLD report proposed to merge GOLD C and D (into “E”) based on “the clinical relevance of exacerbations, independently of the level of symptoms of the patient”.^3^ We demonstrate that symptoms (CAT scores) are critical for stratifying disease progression both in-terms of symptomology and future exacerbation risk. In particular, we find that individuals initially stratified into GOLD C demonstrate a significantly lower moderate and severe exacerbation rate (in 12 months post classification) than those initially in GOLD D. Symptoms (measured by CAT) in the 12-months post GOLD classification remain significantly lower for those initially in GOLD C than GOLD D. As a result, only 25.2% of GOLD C users would escalate to GOLD D at the following 12-month review. This is relative to 38.5% of GOLD C users who could be de-escalated to GOLD A or B after 12-months. Based on this evidence, it is unlikely that users in GOLD C and D would converge after 12-months.

A second motivating factor for a proposed merger of GOLD C and GOLD D was GOLD C representing a relatively small proportion of patients with COPD. While GOLD group C is 6% of our cohort, we note that it represents an older population (Kolmogorov–Smirnov statistic = 0.24; P = 7.0 × 10^−4^) with typically lower smoking rates relative to GOLD D (14.8% of GOLD C; 16.5% of GOLD D). We note that the prevalence of GOLD C would rise to 9.6% for our cohort at the following 12-month review. Despite it being the least frequent GOLD group, it still represents a significant and distinct population which potentially require different treatment plans to GOLD D.

Limitations of this study may include generalisability due to the inclusion criteria. myCOPD is prescribed by a clinician as part of an agreed self-management plan with previous studies suggesting neither age, wealth, nor geographical location represent significant barriers to using the app.^6^ Despite this, we selected users who have registered CAT scores over a 12-month period potentially selecting COPD patients who are more proactive in self-management. The cohort distribution does not seem skewed to lower acuity (ie, exacerbation history) relative to other UK cohorts.^12^

## Conclusion

In conclusion, this analysis highlights that symptomology still can play an important role in the stratification of COPD patients and the GOLD guidelines should re-examine whether the combination of C and D is appropriate. The creation of GOLD E aims to simplify therapy guidance for clinical practice. However, GOLD E will contain a variety of disease acuities, which could potentially lead to a loss of clarity and over-treatment in clinical practice. In future work, we will validate whether myCOPD can support automated review of patient medications relative to GOLD treatment guidelines in order for clinicians to optimise treatment aimed at reducing exacerbation risk.

## Abbreviations

COPD, chronic obstructive pulmonary disease; CAT, COPD Assessment Test; GOLD, Global Initiative for Chronic Obstructive Lung Disease; mMRC, Modified Medical Research Council.

## Data Sharing Statement

Data will be made available upon reasonable request to persons with a university affiliation. Requestors will need appropriate data protection, governance, and ethical review in place.

## References

[1] Rabe KF, Hurd S, Anzueto A, et al. Global strategy for the diagnosis, management, and prevention of chronic obstructive pulmonary disease: GOLD executive summary. *Am J Respir Crit Care Med*. 2007;176(6):532–555. doi:10.1164/rccm.200703-456SO 17507545

[2] Vogelmeier CF, Criner GJ, Martinez FJ, et al. Global strategy for the diagnosis, management, and prevention of chronic obstructive lung disease 2017 report. GOLD executive summary. *Am J Respir Crit Care Med*. 2017;195(5):557–582. doi:10.1164/rccm.201701-0218PP 28128970

[3] Agustí A, Celli BR, Criner GJ, et al. Global initiative for chronic obstructive lung disease 2023 report: GOLD executive summary. *Am J Respir Crit Care Med*. 2023;207(7):819–837. doi:10.1164/rccm.202301-0106PP 36856433 PMC10111975

[4] North M, Bourne S, Green B, et al. A randomised controlled feasibility trial of E-health application supported care vs usual care after exacerbation of COPD: the RESCUE trial. *NPJ Digital Med*. 2020;3(1):145. doi:10.1038/s41746-020-00347-7 PMC7603326 33145441

[5] Crooks MG, Elkes J, Storrar W, et al. Evidence generation for the clinical impact of myCOPD in patients with mild, moderate and newly diagnosed COPD: a randomised controlled trial. *ERJ Open Res*. 2020;6(4):00460–2020. doi:10.1183/23120541.00460-2020 33263052 PMC7682704

[6] Cooper R, Giangreco A, Duffy M, et al. Evaluation of myCOPD digital self-management technology in a remote and rural population: real-world feasibility study. *JMIR mHealth uHealth*. 2022;10(2):e30782. doi:10.2196/30782 35129453 PMC8861861

[7] Rodriguez-Roisin R. Toward a consensus definition for COPD exacerbations. *Chest*. 2000;117(5):398S–401S. doi:10.1378/chest.117.5_suppl_2.398S 10843984

[8] Chmiel FP, Burns DK, Pickering JB, et al. Prediction of chronic obstructive pulmonary disease exacerbation events by using patient self-reported data in a digital health app: statistical evaluation and machine learning approach. *JMIR Med Informatics*. 2022;10(3):e26499. doi:10.2196/26499 PMC8981014 35311685

[9] Dodd JW, Hogg L, Nolan J, et al. The COPD assessment test (CAT): response to pulmonary rehabilitation. A multicentre, prospective study. *Thorax*. 2011;66(5):425–429. doi:10.1136/thx.2010.156372 21398686

[10] Gupta N, Pinto LM, Morogan A, et al. The COPD assessment test: a systematic review. *Eur Respir J*. 2014;44(4):873–884. doi:10.1183/09031936.00025214 24993906

[11] my mHealth Ltd. Privacy Notice. Available from: https://eur03.safelinks.protection.outlook.com/?url=https%3A%2F%2Fmymhealth.com%2Fprivacy&data=05%7C01%7CC.J.Duckworth%40soton.ac.uk%7Cb876fa94a19945e6974008db9506fe81%7C4a5378f929f44d3ebe89669d03ada9d8%7C0%7C0%7C638267629484317669%7CUnknown%7CTWFpbGZsb3d8eyJWIjoiMC4wLjAwMDAiLCJQIjoiV2luMzIiLCJBTiI6Ik1haWwiLCJXVCI6Mn0%3D%7C3000%7C%7C%7C&sdata=9DPflF9LRryfhz0MCsihweODtoTFa%2FTfN6VuWIT49%2BI%3D&reserved=0. Accessed October 17, 2023.

[12] Halpin DM, de Jong HJ, Carter V, Skinner D, Price D. Distribution, temporal stability and appropriateness of therapy of patients with COPD in the UK in relation to GOLD 2019. *EClinicalMedicine*. 2019;14:32–41. doi:10.1016/j.eclinm.2019.07.003 31709400 PMC6833455

